# Social Attention, Affective Arousal and Empathy in Men with Klinefelter Syndrome (47,XXY): Evidence from Eyetracking and Skin Conductance

**DOI:** 10.1371/journal.pone.0084721

**Published:** 2014-01-08

**Authors:** Sophie van Rijn, Marjolein Barendse, Stephanie van Goozen, Hanna Swaab

**Affiliations:** 1 Clinical Child and Adolescent Studies, Leiden University, Leiden, The Netherlands; 2 Leiden Institute for Brain and Cognition, Leiden, The Netherlands; 3 School of Psychology, Cardiff University, Cardiff, United Kingdom; University of Lincoln, United Kingdom

## Abstract

Individuals with an extra X chromosome (Klinefelter syndrome) are at risk for problems in social functioning and have an increased vulnerability for autism traits. In the search for underlying mechanisms driving this increased risk, this study focused on social attention, affective arousal and empathy. Seventeen adults with XXY and 20 non-clinical controls participated in this study. Eyetracking was used to investigate social attention, as expressed in visual scanning patterns in response to the viewing of empathy evoking video clips. Skin conductance levels, reflecting affective arousal, were recorded continuously during the clips as well. Empathic skills, i.e. participants' understanding of own and others' emotions in response to the clips was also assessed. Results showed reduced empathic understanding, decreased visual fixation to the eye region, but increased affective arousal in individuals with Klinefelter syndrome. We conclude that individuals with XXY tend to avoid the eye region. Considering the increased affective arousal, we speculate that this attentional deployment strategy may not be sufficient to successfully downregulate affective hyper-responsivity. As increased affective arousal was related to reduced empathic ability, we hypothesize that own affective responses to social cues play an important role in difficulties in understanding the feelings and intentions of others. This knowledge may help in the identification of risk factors for psychopathology and targets for treatment.

## Introduction

Klinefelter syndrome (KS) is a chromosome aneuploidy characterized by an additional X chromosome in boys and men (47, XXY), with a prevalence of 1∶500 to 1∶1000 male births [1]. The presence of an exceptional high density of genes on X chromosome that are essential for neural development [2], warrants the study of cognitive functioning in individuals with an extra X chromosome. Intellectual level of boys and men with KS is typically in the normal range, although on average somewhat lower, specifically with regard to verbal IQ [3], [4]. However, there is an increased risk for specific cognitive dysfunctions in areas of language, executive functioning and social cognition [3]–[8].

Social and emotional functioning of individuals with KS has recently received increased attention. Although the behavioural phenotype may be variable, on average boys and men with KS are at risk for difficulties with social interactions and social adjustment. These social difficulties include shyness, social withdrawal, social anxiety, difficulties in peer-relationships, social impulsivity, communication difficulties, depressed adaptive skills, and reduced social assertiveness [9]–[16]. Also, recent studies have suggested that boys and men with Klinefelter syndrome are at increased risk for autism traits and symptoms [13], [17]–[20].

Traditionally, it has been thought that social dysfunction arises from the language deficits typically seen in males with KS or it has been considered a consequence of daily life struggles associated with the condition. However, recent studies have shown that impairments in social cognitive processing may also play an important role. Social cognitive processing, i.e. the ability to perceive, understand and express social signals, has a contribution to social functioning that is independent of other cognitive abilities such as intelligence, language and attention [21]. There is evidence showing that Klinefelter syndrome can be associated with increased risk for difficulties in the identification of emotions of others based on facial expressions [7], difficulties with recognizing affective tone of voice (emotional prosody) [22], and reduced sensitivity to gaze direction [8]. Interstingly, there are also data suggesting that Klinefelter syndrome can be associated with abnormalities in emotional reactivity. In earlier studies using self-report measures, men with KS reported to be more easily affectively aroused than controls in response to emotion inducing events [7], and reported to experience higher levels of distress than controls during social interactions, for example when dealing with criticism or when starting a conversation with others [13]. These studies on social and emotional processing call for further investigation, particularly considering social dysfunctioning and vulnerability for autism symptoms in individuals with Klinefelter syndrome. The present study aims to provide in this by examining to what degree Klinefelter syndrome is associated with abnormalities in a) empathy in response to social stimuli, b) attention towards social stimuli and c) affective (physiological) arousal in response to social stimuli.

Empathy has been defined as ‘the capacity to (*a*) be affected by and share the emotional state of another, (*b*) assess the reasons for the other's state, and (*c*) identify with the other, adopting his or her perspective’ [23]. Baron-Cohen emphasized that empathic responses entail appropriate emotions, that are triggered by someone else's emotional state [24].Empathy is essential for the regulation of social interactions, as it allows one to automatically and quickly relate to the emotional states of others [23]. Because empathy allows one to understand the feelings and intentions of others, it helps in predicting their behavior [24]. It has been proposed that limited empathy is one of the neurocognitive constructs underlying the social and communicative difficulties observed in individuals with ASD [25]. Empathy has not yet been studied in individuals with Klinefelter syndrome.

Social attention involves an spontaneous and automatic visual orientation towards objects with social importance, namely individuals, their faces, and especially their eyes [26]. Due to the extensive amount of information that can be extracted, the eye region represents a crucial area of the face. More than other facial features, the eyes are central to all aspects of social communication such as identity, emotional state as well as direction of attention and related potential targets for intentions. This is also illustrated by studies showing that scanning of the face always starts with the eyes regardless of its emotion [27], [28]. A review by Itier [29] demonstrated that of all facial features the eye region is the most attended to and the source of information most utilized, independent of the task, whether it concerns gaze, head orientation, identity, gender, facial expression or age. Considering that individuals with autism spectrum disorders (ASD) often show a reduced tendency to focus on the eyes of others, which has been related to impairments in understanding the emotions of others and increased social anxiety [30], [31], a study on attention to such social cues in individuals with Klinefelter syndrome is warranted.

Affective arousal is an indicator of emotional reactivity. An emotion is ‘a complex psychological state that involves three distinct components: a physiological response, a subjective experience (feeling), and a behavioral or expressive response’ [32]. For social adaptation, the regulation of emotions plays an important role. Emotion regulation refers to the ability to monitor, evaluate, and modify the intensity and temporal dynamics of emotional reactions through extrinsic means (managing overt behaviors and social situations) and intrinsic means (recruiting cognitive and psychophysiological systems) [33], [34]. Emotions help facilitate behaviors that are adaptive to the social environment [33]. However, emotions can also negatively impact social functioning of an individual when emotions occur at the wrong time, in the wrong place and at the wrong intensity level. Studies on ASD suggest abnormalities in the intensity level of affective arousal to social stimuli [35], which may result from emotion regulation impairments [36]. Interestingly, Dalton, et al [37] showed that in individuals with ASD increased affective arousal, as reflected in increased neural activation in the amygdala, is associated with reduced gaze fixation. They proposed that reduced fixation towards eyes of others as seen in individuals with ASD, may help downregulate this overarousal to social cues.

So far, studies on emotional reactivity in individuals with Klinefelter syndrome primarily included behavioural measures and self-report data; no research has been done on psychophysiological indices of affective arousal, i.e. implicit, objective arousal responses that may or may not match with explicit, subjective experience of emotion. One of the psychophysiological indices of affective arousal is skin conductance (SC). SC changes are mainly dependent on the activity of sweat glands innervated by the sympathetic branch of the autonomic nervous system (ANS), which is involved in up- and down-regulation of many homeostatic mechanisms in the body, preparing organisms for ‘flight or fight’ responses. The most frequently used measures of ANS activity, reflected in changes in the electrical properties of the skin, are the number of fluctuations in skin conductance (skin conductance responses, SCRs), the amplitude of these fluctuations, and the overall tonic level of the skin conductance. Skin conductance is a sensitive and objective index of implicit emotional responses that may occur without conscious awareness or are beyond cognitive intent [38], [39]. In the general population, the presentation of affective faces or scenes typically leads to a short increase in skin conductance, reflecting an affective response [40], [41]. Emotional video clips typically also induce physiological reactions, such as an increase in skin conductance level and in amount and amplitude of SCRs, reflecting affective arousal [42]. The strength of skin conductance responses varies from person to person.

To summarize, individuals with KS often have problems in social and emotional functioning, and are at higher risk for autism traits. In the search for underlying mechanisms driving this increased risk we propose that the study of social cognition and emotion processing, in addition to language and other cognitive domains, may help in the identification of risk factors and targets for treatment. In this study we focused on social attention and emotion processing in KS using a neurocognitive approach. To this end, we investigated physiological arousal (i.e., skin conductance level) and visual scanning patterns whilst viewing empathy evoking video clips. These implicit measures of social attention (fixation patterns) and emotional reactivity (arousal) were related to more explicit measures, i.e. the understanding of emotions of others and one's own emotions in response to the video clips.

## Methods

### Ethics statement

The study was approved by the Ethics Committee of Leiden University. Written informed consent, according to the declaration of Helsinki, was obtained from all participants.

### Participants

Seventeen (young) adults with Klinefelter syndrome (mean age 37.91 years, SD 12.02) were included in this study, as well as 20 non-clinical controls (mean age 32.64 years, SD 12.23). Mean age did not differ between groups, but the mean education level was higher for the control group (*F*(1,35) = 26.72, *p*<0.001). Participants with KS were recruited with help of the Dutch Klinefelter support group (NKV) or were attending the outpatient clinic at the department of Child and Adolescent Studies at Leiden University. Seven of the men with XXY were using testosterone supplements. Control participants were recruited by the researchers, with help of information brochures that were distributed in public areas outside university campus. All participants had normal or corrected-to-normal vision. In the XXY group, mean IQ was 94.21 (SD 14.81). Written informed consent, according to the declaration of Helsinki, was obtained from all participants.

### Intellectual functioning

All participants completed the subtests Blockdesign (perceptual organization skills) and Vocabulary (verbal skills) of the of the Dutch adaptations of theWAIS-III (WechslerAdult Intelligence Scales) [43], [44], i.e. V-BD short form. This short form can be used to estimate full scale IQ (FSIQ) according to the algorithm (2.9*(sum of normed scores)+42) [45]. The V-BD short form correlates high with full scale IQ (*r* = .88) [46] and the V-BD short form has been found valid for the estimation of intelligence, with a good reliability (*r* = .91) and validity (.82) [45].

### Eye tracking during affective clips

After a 5 point calibration procedure four affective, empathy evoking video clips of approximately 90 seconds each [47] were presented in randomized order on a LCD screen 65 cm away from the participant. Total fixation duration within specific areas of interest was measured with the Tobii T120 eye tracker (Tobii Technology, Sweden), which records the X and Y coordinates of participant's eye position at 120 Hz by using corneal reflection techniques. Gaze data were processed using Tobii Studio (3.0), using the Tobii I-VT fixation filter. The ‘Dynamic AOI (Area of Interest)’ tool, using freeform shapes, was used to draw AOI's. Dynamic AOIs were grouped into the following categories: *facial features (without mouth and eyes), eyes, mouth, body, objects* and *other (i.e. on the screen, outside other AOI's)*.

The primary emotions of the main character in the video clip were pain/surprise, sadness/upset, fear/anxiety or happy/cheerful, based on ratings obtained in a pilot study aimed at identifying the two most reported emotions for each video clip. The four video clips were scenes selected from t.v. broadcasted movies. The ‘pain/surprise’ clip concerned a man getting stuck between rocks while climbing, and showed his face while amputating his own arm (not shown) in order to survive. The ‘sad/upset’ clip showed the reaction of a boy who is told his father has just died. The ‘fear/anxiety’ clip showed a child in a rowing boat at sea, while faced with a shark attack. The ‘happy/cheerful’ clip showed the ecstatic reaction of a horse racer en his team while winning the race. In all clips, auditory input was included, as vocal responses (including sounds of pain and crying as well as affective prosody, i.e. emotional tone of voice) are part of social cues in social situations. The clips were English spoken.

There was a fixed time interval between presentation of the video clips during which the explicit empathy test was performed. Eyetracking data from the four emotional video clips were collapsed for data analysis as total duration of the AOI's within each emotional video was not explicitly controlled for.

### Physiological arousal during affective clips

Physiological data were collected continuously during the entire video presentation with a sample rate of 500 samples/s using Acqknowledge software (Biopac System Inc.). Measurements were acquired through a Galvanic skin response amplifier (GSR100C) and a Biopac data acquisition system (mp150-Windows). The physiological monitoring equipment was synchronized with Tobii software by event markers representing the beginning and ending of each video. In Acqknowledge, a 0.5–2 Hz band-pass filter was applied. A skin conductance response was defined as an increase in Skin conductance level (SCL) with a minimum of 0.05 µS within a timeframe of 3 seconds (considering the typical rise time for a skin conductance response of 1 to 3 seconds). SCL ( µS) and number of skin conductance responses (SCRs) per minute were examined. Skin conductance measurements were done during a rest condition and during the four emotional video clips. During the non-emotional, rest measurement participants watched a relaxing video clip of an aquarium, lasting three minutes.

### Explicit empathy test

After each clip participants completed two questionnaires, one concerning the emotions of the main character and the other concerning their own emotions while viewing the clip. By placing a cross on a continuous line of 10 cm they indicated how strong they or the main character felt on a range of 10 emotions (anger, sad, pain, upset, fearful, happy, scared, cheerful, surprised and hurt). Participants were also asked to explain the reason for the emotion they rated that they felt most strongly. A total empathy score was calculated, based on three components: recognizing the most important emotion of the main character (there were two emotions for each clip that were considered ‘correct’ based on a pilot study at Cardiff University), feeling emotions concurrent with the emotions of the main character and giving an empathic reason for one's own emotion(s), similar to Braaten, et al. [48], although they computed separate scores for matching of emotions and interpretation of emotions. Total empathy score in this study was calculated as follows. If participants gave the two ‘correct’ emotions of the main character a higher score (i.e. stronger intensity) than the other emotions on the list, they received two points (one point per emotion). Furthermore, they received two points if the emotions they indicated as strongest and second strongest for themselves, were the same emotions as those which they indicated as strongest and second strongest for the main character (one point for each emotion). Finally, they received a point if the reason they gave for their own emotion(s) was empathic, defined as ‘The participant provided an explanation that included a direct reference to the character's feelings or experience’. In addition, explanations for own emotions should fit the content of the video clip, in order to count as an empathic response. For example, an empathic response would be when participants stated they wondered what it would be like for him/her to experience that. An example of a non-empathic reason would be “I was surprised to see this movie”. This leads to a maximum score of 20 points, 5 for each video. The reasons participants gave were coded afterwards by the experimenter and a second coder who was (in contrast to the experimenter) blind to the clinical status of the participants. Interrater agreement was 92.6%.

### Procedure

Before electrodes were attached, participants cleaned their hands. Skin conductance electrodes were placed on the middle phalanges of the ring and middle fingers of the participant's non-dominant hand. A non-affective (baseline) video clip was shown first, after which the 4 emotional video clips were presented in counterbalanced order. Before the start of the non-affective clip participants were instructed to sit quietly, watch the video and try to relax. Before the start of the empathy evoking video clips, participants were instructed to watch the four short clips, sit quietly and minimize their movements, and answer the questions subsequently.

### Statistical analyses

Total empathy scores in the two groups (control, XXY) were compared using ANVOCA, controlling for verbal skills. For groupwise (XXY, control) comparisons in specific empathic scores (3 subscales) and fixation duration (6 AOI's), MANCOVA and ANCOVA was used respectively. To exclude that verbal skills had an effect in the performance test, normscore on the Vocabulary subtest of the WAIS-III was included as a covariate in analyses of the explicit empathy test. Repeated measures analysis was used to assess group differences in changes in SCL and SCRs from rest to the emotional video clips (4 clips). Only those variables that showed significant group differences (control, XXY) were included in correlational analyses. For correlational analyses, spearman's rho was used. Level of significance was set at p<0.05.

## Results

### Intellectual functioning

Mean IQ was 94.22 (SD 14.81) in the XXY group and 108.14 (SD 10.33) in the control group, which was significantly different (F(1,35) = 11.21, p = 0,002, but within the normal range for all participants. Mean normscore for the subtest Vocabulary was 8.03 (SD 2.12) for the XXY group and 12.42 (SD 2.01) for the control group, which was significantly different (F(1,35) = 42.02, p<0.001. Mean normscore for the subtest Blockdesign was 10.05 (SD 3.64) for the XXY group and 10.44 (SD 2.65) for the control group, which was not significantly different (F(1,35) = 0.11, p = 0.74.

### Explicit empathy test

The total empathy score was 13.01 (*SD* 2.45) in the control group and 9.44 (*SD* 2.83) in the XXY group, which was significantly different (*t*(1,34) = 9.31, *p* = 0.004, Cohen's *d* = 1.44) as indicated by ANCOVA (controlling for verbal skills).

**Figure 1:**
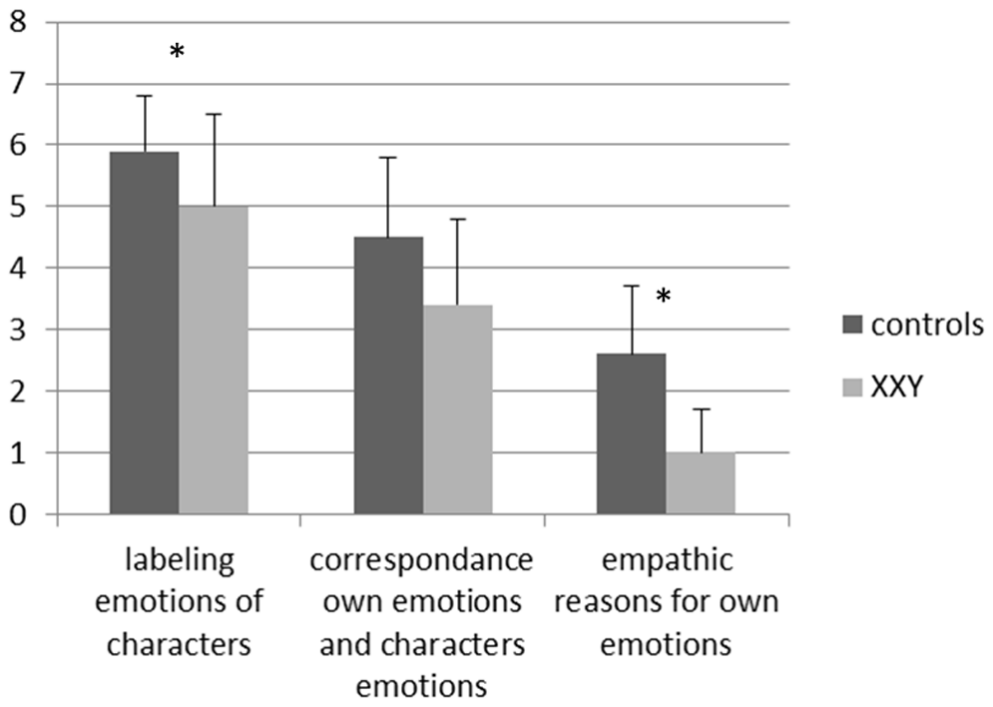
Empathy scores (mean, SD) in the XXY group versus the control group. *significant at p = 0.05.

MANCOVA with the three empathy scores showed a main multivariate effect of group (control, XXY), F(3,32) = 4.94, p = 0.006, but no main multivariate effect on the covariate verbal skills (p = 0.70). Univariate effects of group were present for correctly labelling the emotions of the main characters in the affective clips (*F*(1,34) = 4.32, *p* = 0.04, Cohen's *d* = 0.73), and empathic explanations for their own emotions (*F*(1,34) = 12,02, *p* = 0.001 Cohen's *d* = 1.70), with lower scores in the XXY group. However, there was no significant effect of group for correspondence between their own reported emotions and the emotions of the main characters (*p* = 0.17). In the XXY group there were no systematic confusions in terms of mislabeling emotions. Data are presented in figure 1.

### Social attention during empathy clips: Eyetracking

**Figure 2:**
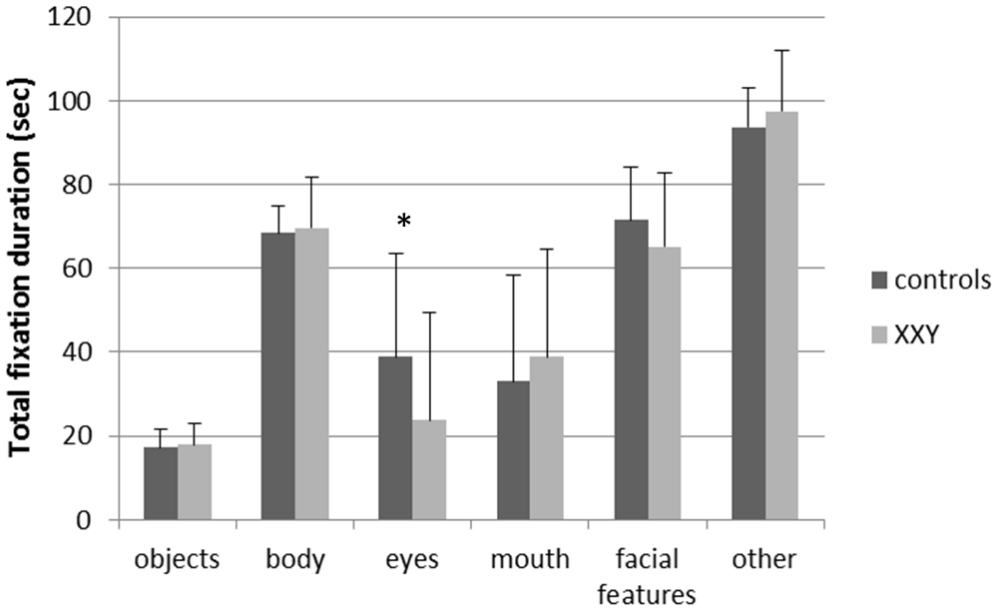
Total fixation duration (mean, SD) within specific areas of interest (AOI) while watching emotional video clips for the XXY group and control group separately. *significant at p = 0.05.

**Figure 3:**
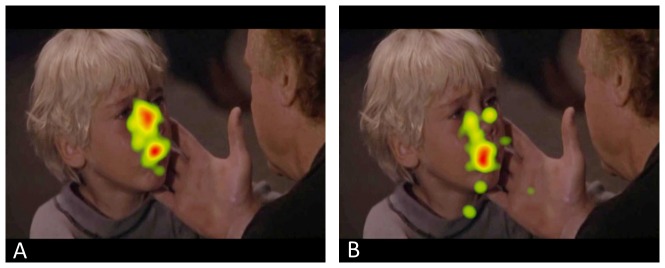
Heat maps of the control group (A) en XXY group (B) reflecting total fixation duration in a scene involving a boy being told his father has died, illustrating the group differences in visual fixation on the eyes.

ANOVA indicated a significant group effect on total fixation duration in the AOI ’eyes’ (*F*(1,35) = 4.50, *p* = 0.04. The XXY group spend less time fixating on eyes as compared to the control group, with an effect size of 1.23 (Cohen's *d*). This appeared to be a specific effect, as other AOI's did not show significant group effects. Covarying for level of education did not change the significance of the results. Furthermore, within the XXY group, there were no significant differences in fixation duration between those who were using and not using testosterone supplements. For mean total fixation durations in each AOI, see figure 2. An illustration of eyegaze patterns in both groups is presented in figure 3.

### Relation between visual scanning patterns and self-reported empathy

Within the XXY group and the control group, there were no significant correlations between total fixation duration to the eyes and the empathy scores.

### Physiological arousal during affective clips: Skin conductance

**Figure 4:**
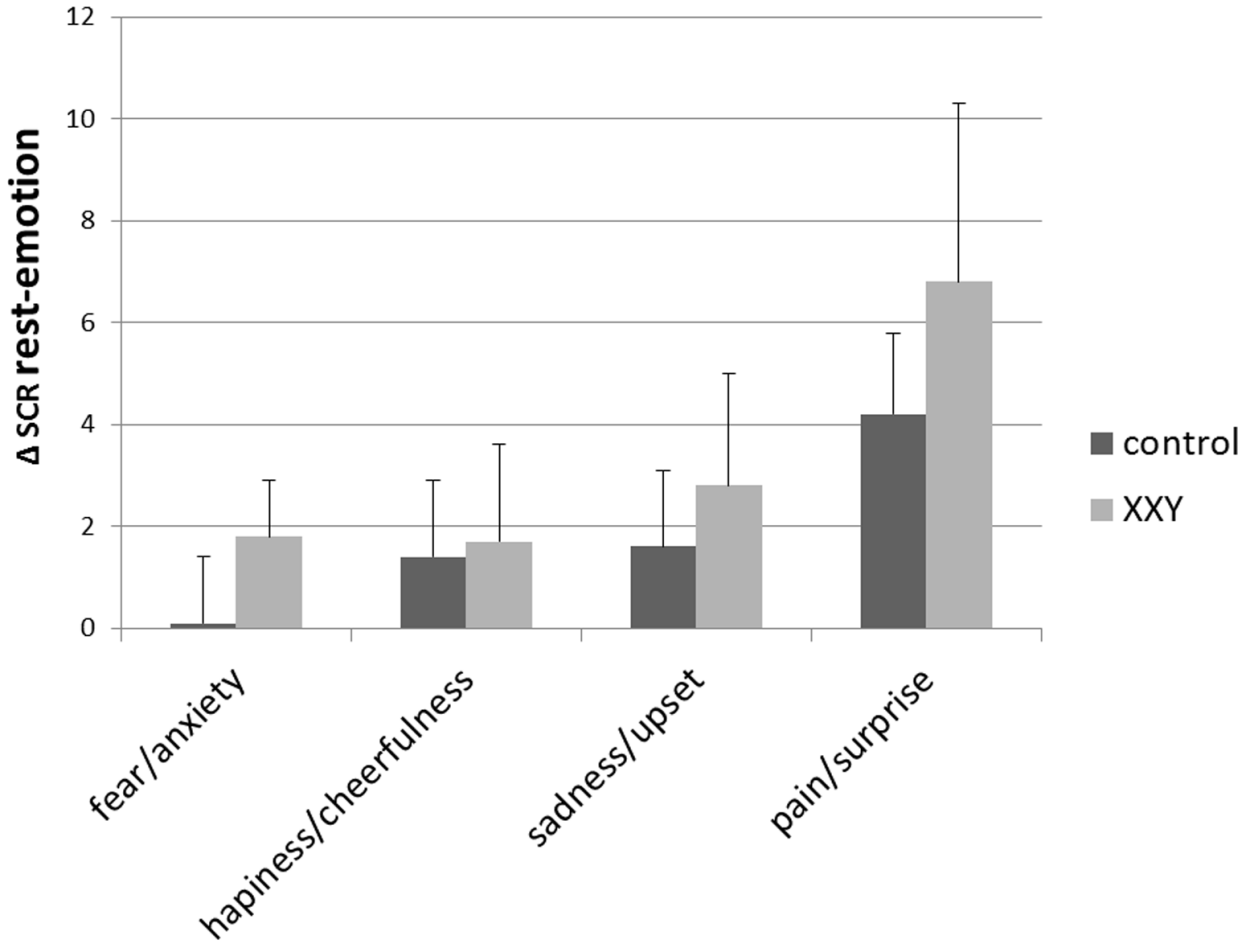
Increase in number of SCRs (mean, SD) per minute from rest to the affective clips in the control group and XXY group. * significant at p<0.05.

One outlier, with a *Z* score of 3.84 for skin conductance level during rest, was removed from the control group. We analyzed both skin conductance level (SCL) and the number of skin conductance responses per minute (SCRs). During the rest condition, while viewing the non-emotional video clip, there was a significant difference between the control group and XXY group in SCL (*F*(1,34) = 7.82, *p* = 0.008) and SCR (*F*(1,34) = 10.71, *p* = 0.002), with lower skin conductance levels and a lower number of SCRs in the XXY group. Considering that many factors influence absolute levels of skin conductance, our main interest was in a relative measure, i.e. group differences in affective responsivity. For this, we used repeated measures analyses to compare the increase in arousal from rest to the emotional clips in both groups. All repeated measures analyses showed a significant main effect of condition (at p<0.01), indicating that arousal (expressed in SCL and SCRs) was overall higher during the affective clips as compared to rest (irrespective of group membership). For SCRs, a significant group (control, XXY) by condition (rest, emotion) interaction was found for ‘fear/anxiety’ (*F*(1,34) = 5.82, *p* = 0.02) and ‘pain/surprise’ (*F*(1,34) = 4.43, *p* = 0.04). The increase in arousal from rest to the affective clips (i.e. affective responsivity) was larger in the XXY group as compared to the control group. The differences in SCRs between rest and the affective clips are presented for both groups in figure 4. For SCL, no significant group (control, XXY) by condition (rest, emotion) interactions were observed.

### Relation between physiological arousal and self-reported empathy

Within the XXY group, the number of SCRs during the fear/anxiety video clip was significantly inversely correlated with the total empathy score (*r* = −0.53, *p* = 0.02). In others words, higher levels of arousal during the fear/anxiety video clip were related to lower levels of empathy. Importantly, the number of SCRs during rest was not correlated with total empathy score (*r* = 0.01, *p* = 0.94), indicating that the effect was specific for the emotional video clip. In the control group, there was no significant correlation.

## Discussion

In this study we investigated empathy, social attention, and affective arousal in response to social stimuli in individuals with Klinefelter syndrome (KS). Social attention (eye gaze patterns) and emotional reactivity (physiological arousal) were measured and related to more explicit, behavioral measures, i.e. the understanding of own and others' emotions.

The behavioral measures showed that individuals with KS compared to controls had more difficulty responding in an empathic way to the emotional video clips, as reflected in an overall lower empathy score, which was driven by a reduced ability to identify the emotions of the main character and in showing less empathic reasons they reported for their own emotion(s). There were no significant group differences in rating own emotions in concordance with the main character's emotions. The empathy deficits were independent of verbal skills, as this was controlled for in the analyses, and which showed no significant effect of verbal skills on empathy scores. In the XXY group, mean total empathy score was 1.44 standard deviations below that of controls.

Objective measures of social attention revealed atypical eyegaze patterns in the XXY group. Eyetracking during viewing of the emotional video clips showed that individuals with KS spent less time looking at the eyes of the video characters as compared to the control group (i.e. displayed a shorter fixation duration). The effect size (Cohen's *d*) indicated that mean fixation duration in the XXY group was 1.23 standard deviations below that of controls. This effect was specific for the eye region, as looking times for other type of stimuli such as ‘face’, ‘body’, ‘object’ and ‘mouth’ were similar for the KS and control groups. Looking time outside these areas but on screen was also similar, indicating that there was no overall difference in attention to the video clip. Taken together, considering that in social communication the most crucial information comes from the eyes, a reduced tendency to focus on the eyes of others in individuals with KS can have profound effects on adaptive social functioning. The related deficits in empathic understanding in KS support this interpretation.

Support for the link between social attention impairments and social dysfunction largely comes from studies in the field of autism. For example, when looking at video clips of social situations, individuals with autism spectrum disorder (ASD) spend less time focusing on eyes and more time focusing on mouths, bodies and objects [49]. Differences in eyegaze patterns have also been found when individuals with and without ASD view pictures of faces [37], [50], with reduced attention to the eye region in those with ASD. Several studies have found that in individuals with autism less attention to the eye region of faces is associated with more impaired emotion recognition skills [30], [31] and higher levels of social anxiety [31]. Another study found similar relations in children with ASD and typically developing controls: emotion recognition accuracy was positively related to fixation time on the eye region and negatively to fixation time on the mouth region [51]. We speculate that reduced attention to the eye region, and related difficulties in empathy and emotion understanding, might in part drive the increased vulnerability for social difficulties and autism traits in individuals with KS.

Besides difficulties in processing of emotions of others, we also observed abnormalities with regard to own emotions in adults with KS. To study emotional reactivity, we used an objective measure of affective arousal as expressed in psychophysiological responses, i.e. skin conductance, during the emotional video clips. This showed that in comparison to the control group, individuals with KS showed a stronger increase in arousal response to emotional video clips compared to controls. This was specifically observed for the clips in which the main character was experiencing fear/anxiety or pain/surprise. This finding of increased affective responsivity matches up with earlier behavioural studies. Using a behavioural questionnaire, Ratcliffe, et al [14] found that a group of 14 boys with KS were on average ‘more affected by feelings’ than the control group. In a study on emotion processing using self-report questionnaires, men with KS reported to be more easily emotionally aroused by emotion inducing events (‘emotionality’) as compared to the control group [7]. Also, increased experience of distress during social interaction was reported (using questionnaires) by adults with KS in a study on social functioning and autism traits [13].

Interestingly, higher levels of affective arousal in the XXY group were associated with lower overall levels of empathic ability, as reflected in a reduced ability to identify the emotions of the main character, their own emotions being less concordant with the main character's emotions and in showing less empathic reasons they reported for their own emotion(s). A decreased ability to identify and label emotions was not only evident in this study, but also observed in an earlier behavioural study [7] showing that men with KS reported a reduced ability to identify, experience, verbally describe and reflect on one's own affective arousal states, which also been conceptualized as ‘alexithymia’ (literally translated ‘lacking words for feelings’) [52], [53]. Such a mismatch between reduced (subjective) labeling of emotion versus increased (objective) affective arousal is shown to be associated with deficient emotion regulation [54]. Although speculative, the degree of affective arousal in response to social cues may (in part) reflect emotion regulation deficiencies as has been suggested for individuals with ASD [36].

The emotion regulation model by Gross [34] distinguishes antecedent focused emotion regulation mechanisms and response focused emotion regulation mechanisms, with some more adaptive than others. Response-focused regulation occurs after the emotional responses are generated. It includes response modulation, which refers to efforts to suppress the experience or expression of emotion. Antecedent regulation is obtained by (1) altering the interpretation of a situation to change its emotional impact, i.e. cognitive change or (2) directing attention towards or away from specific aspects of the situation, i.e. attentional deployment. The two key attentional deployment mechanisms described by the process model are *concentration* and *distraction*. Distraction involves changing the focus of attention to different aspects of the situation or moving attention away from the situation altogether. It may also involve a change in internal focus, such as invoking memories or thoughts that are inconsistent with the undesirable emotional state. Based on our finding of less attentional focus to the eye region in individuals with KS (interpreted as avoidance), we speculate that individuals with KS use attentional deployment, specifically distraction, as an emotion regulation mechanisms. However, as participants were not instructed to exert effortful regulation of their emotions, our findings may largely reflect implicit and automatic attentional deployment. In contrast to explicit use of emotion regulation strategies, requiring conscious effort, monitoring, and some level of insight or awareness, emotion regulation mechanisms can also be triggered automatically by the stimulus itself and can take place without monitoring, insight or awareness [55].

A recent meta-analysis has shown that strategies involving attentional deployment prove to be less effective (effect size 0.0) than strategies involving cognitive change, i.e. reappraisal, which had a small-to-medium sized effect (effect size 0.36) on emotional responses [56]. Although speculative, the use of a less effective emotion regulation strategy does line up with the finding of increased emotional arousal in the XXY group. Interestingly, Bebko, et al. [57] using eyetracking found that irrespective of emotion regulation strategy, individuals who looked less at emotional areas of presented pictures, were less likely to experience emotion regulation success.

Although picking up emotional signals of others is important for successful social interactions [58], [59], one's own emotions seem to play an equally important role in social adaptive behavior [60], [61]. Poor emotion regulation has been associated with lower quality of friendships, reduced interpersonal sensitivity, less prosocial tendencies and more social conflicts in young adults [60]–[62] as well as reduced social adaptation and low peer friendship nominations in children and adolescents [63]–[65]. In line with our findings, deficiencies in affective reactivity and identification and labeling of affect have also been reported in the field of autism. High levels of alexithymia, including difficulties in adequately assessing and labeling own affective arousal states, have been observed in individuals with ASD [66]–[71]. Interestingly, children and adolescents with ASD have also been found to show a stronger skin conductance reaction to faces with direct and averted gaze compared to typically developing children [35]. However, findings are inconsistent as other studies have found that children and adults with ASD show similar skin conductance responses to objects and faces, in contrast to individuals without ASD who show a stronger reaction to faces than to objects [72], [73].

This study also had several limitations, which should be addressed. First, the XXY group may not be representative for KS individuals at large, considering that inclusion was done in a subgroup who was seeking information about their condition or seeking help for psychological problems. Nonetheless, findings in this article are applicable and relevant for those individuals with KS who have social difficulties. Second, the sample sizes were relatively small, based on which one should be cautious in interpreting and generalizing the results. Especially for the correlational analyses, our findings need replication in a larger sample. Third, we were not able to compare visual fixation duration across emotions, as exposure times of the areas of interest were not explicitly controlled for across the video clips. Finally, participants were not explicitly instructed to regulate their emotions during viewing of the video clips, so we were not able to assess explicit emotion regulation skills.

**Figure 5:**
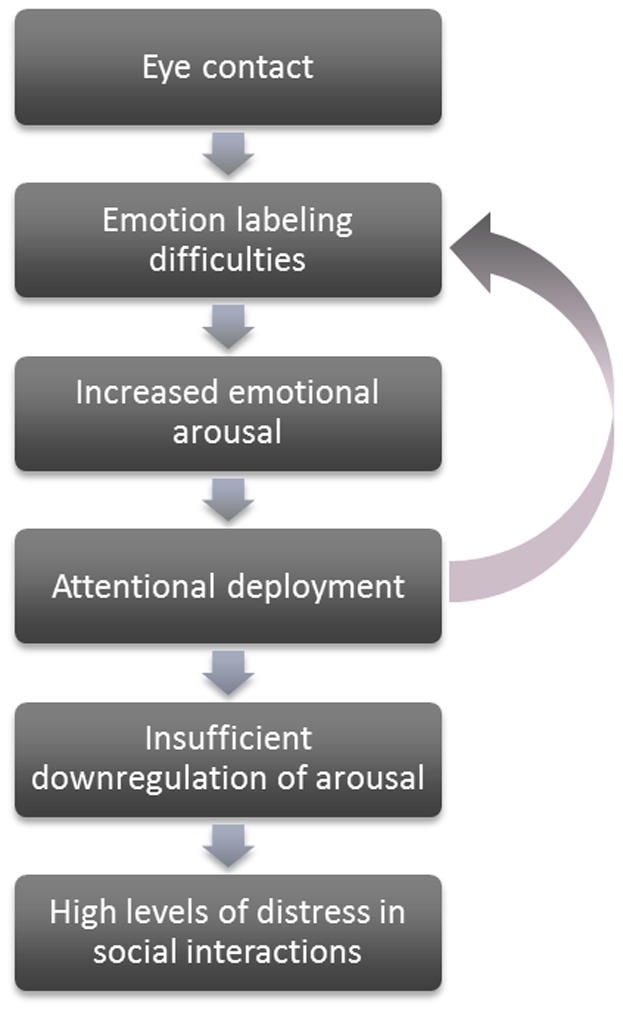
The emotion regulation hypothesis of social dysfunction in Klinefelter syndrome.

Taken together, our data suggest a profile of increased emotional arousal, together with decreased attention to and difficulties with labeling of emotional information, in individuals with Klinefelter syndrome. These findings tentatively suggest impairments in emotion regulation and social attention. As increased affective responsivity was related to reduced empathic ability, we hypothesize that emotional responsivity plays an important role in difficulties in understanding the feelings and intentions of others. Although speculative, focussing on the eyes of others might evoke increased emotional arousal in individuals with KS, because of the difficulties in emotion labelling. As a result, attention may be automatically diverted away from the eyes. However, as attentional deployment has proven to be less effective in the regulation of emotions than reappraisal strategies, this may not be sufficient to successfully downregulate this increased arousal, resulting in high levels of distress in social interactions. By diverting attention away from the eyes, individuals with KS may also miss out on crucial information with regard to the feelings and intentions of others, which may further add to the emotion labelling difficulties. For an overview of this theoretical model, see figure 5.

Our interpretation is supported by findings in autism, showing that individuals with ASD often show a reduced tendency to focus on the eyes of others, which has been related to impairments in understanding the emotions of others [30]. Also, difficulties in processing own emotions, as expressed in high levels of alexithymia, are related to deficits in joint-attention (i.e. shared eye contact), theory of mind and empathic ability [74], [75] in individuals with ASD. Interestingly, although Theory of Mind is typically considered as the ability to assess other people's mental states, there is compelling evidence that the processes of assessing other's mental states and one's own mental states are closely related [76]. As argued by Samson, et al. [77], this may not only hold for cognitive states, but also for emotional states. Future studies are needed to address the relationship between the processing of own emotions and Theory of Mind skills. It would also be interesting to further study the role of language deficits in relation to empathy impairments and more thoroughly study systematic biases in emotion labeling. Although biases were not observed in the current study, it would be interesting to assess such biases more rigorously, using reaction time paradigms and facial stimuli varying in degree of emotional expression as well as blended emotional expressions.

Understanding the relation between cognition and emotion, and their impact on social adaptation, may help the diagnosis and treatment of individuals with KS. The findings in this study may also contribute to the identification of cognitive and emotional mechanisms that can be targeted in treatment and are aimed at improving social functioning. For example, we speculate that interventions focusing on improving emotion awareness and emotion regulation might also have positive effects on social cognition, as more socially relevant information will be available when adequate emotional responsivity allows for more thorough visual scanning of eyes and other crucial facial features. In turn, any improvements in labeling of social cues that may arise from more thorough visual scanning, might contribute to more adaptive levels affective arousal. Interestingly, it has already been shown that coherence between subjective and objective (psychophysiological) aspects of emotion is greater in those who have specialized training involving the promotion of greater body awareness [78]. Considering the increased vulnerability for psychopathology in KS, further research is needed on the predictive value of emotion regulation deficits and deviant social attention in relation to risk for autism symptoms, as well as other psychopathology such as psychotic symptoms, depression and anxiety. Insight in such underlying mechanisms of behavior, and opportunities to positive influence these mechanisms over the course of development, might also contribute to new insights into etiology and treatment of developmental psychopathology. Future, preferably longitudinal, studies including childhood populations may help in meeting this aim. Such studies may also help in unfolding the developmental course of emotion regulation impairments and assessing the contribution of social cognitive deficits, language impairments as well as executive dysfunctioning.
